# Linking haploinsufficiency of the autism- and schizophrenia-associated gene *Cyfip1* with striatal-limbic-cortical network dysfunction and cognitive inflexibility

**DOI:** 10.1038/s41398-024-02969-x

**Published:** 2024-06-14

**Authors:** Josephine E. Haddon, Daniel Titherage, Julia R. Heckenast, Jennifer Carter, Michael J. Owen, Jeremy Hall, Lawrence S. Wilkinson, Matthew W. Jones

**Affiliations:** 1 Neuroscience and Mental Health Innovation Institute, Cardiff University, Cardiff, UK; 2 Division of Psychological Medicine and Clinical Neurosciences (DPMCN), School of Medicine, Cardiff University, Cardiff, UK; 3 School of Physiology, Pharmacology & Neuroscience, University of Bristol, Bristol, UK; 4 Centre for Academic Mental Health, Population Health sciences, University of Bristol, Bristol, UK; 5 MRC Centre for Neuropsychiatric Genetics and Genomics, Cardiff University, Cardiff, UK; 6 School of Psychology, Cardiff University, Cardiff, UK

**Keywords:** Schizophrenia, Learning and memory

## Abstract

Impaired behavioural flexibility is a core feature of neuropsychiatric disorders and is associated with underlying dysfunction of fronto-striatal circuitry. Reduced dosage of *Cyfip1* is a risk factor for neuropsychiatric disorder, as evidenced by its involvement in the 15q11.2 (BP1–BP2) copy number variant: deletion carriers are haploinsufficient for *CYFIP1* and exhibit a two- to four-fold increased risk of schizophrenia, autism and/or intellectual disability. Here, we model the contributions of *Cyfip1* to behavioural flexibility and related fronto-striatal neural network function using a recently developed haploinsufficient, heterozygous knockout rat line. Using multi-site local field potential (LFP) recordings during resting state, we show that *Cyfip1* heterozygous rats (*Cyfip1*^+/−^) harbor disrupted network activity spanning medial prefrontal cortex, hippocampal CA1 and ventral striatum. In particular, *Cyfip1*^+/−^ rats showed reduced influence of nucleus accumbens and increased dominance of prefrontal and hippocampal inputs, compared to wildtype controls. Adult *Cyfip1*^+/−^ rats were able to learn a single cue-response association, yet unable to learn a conditional discrimination task that engages fronto-striatal interactions during flexible pairing of different levers and cue combinations. Together, these results implicate *Cyfip1* in development or maintenance of cortico-limbic-striatal network integrity, further supporting the hypothesis that alterations in this circuitry contribute to behavioural inflexibility observed in neuropsychiatric diseases including schizophrenia and autism.

## Introduction

Low gene dosage of cytoplasmic FMRP interacting protein 1 (*CYFIP1*) is a candidate risk factor for neuropsychiatric disease by virtue of its involvement in the pathogenic 15q11.2(BP1–BP2) Copy Number Variant (CNV). Deletion of 15q11.2(BP1–BP2) leads to a two to fourfold increase in the risk for schizophrenia, autism and intellectual disability [1–5]. The deletion contains four genes: non-imprinted in Prader–Willi/Angelman syndrome 1 (*NIPA1*) and 2 (*NIPA2*) genes, *CYFIP1* and tubulin gamma complex associated protein 5 gene (*TUBGCP5*) [6]. All are expressed in the brain and may be of relevance to psychopathology, however *CYFIP1* haploinsufficiency is likely to be a significant contributor to the 15q11.2(BP1–BP2)del psychiatric phenotype due to its known involvement in several key brain plasticity-related functions such as dendritic spine morphology and branching [7, 8], and the interaction between *CYFIP1* and fragile X mental retardation 1 (*FMR1*) [9]. Fragile X syndrome is caused by mutations of *FMR1* and is associated with intellectual disability and a variety of psychiatric symptoms [10].

Consistent with these neurobiological inferences, recent work has shown impaired reversal of visual discrimination in a touchscreen task in a novel rat model haploinsufficient for *Cyfip1* [11]. This inability to respond flexibly was linked to white matter changes observed using diffusion tensor imaging (DTI), that were similar to those seen in 15q11.2(BP1–2)del CNV carriers [12] and *Cyfip1*-deficient mice [13]. In all three species, marked changes to white matter were seen in corpus callosum microstructure, consistent with previous findings reported by Ulfarsson [5], and with reduced callosal volume observed in patients with schizophrenia [14].

White matter abnormalities in frontostriatal loops have been associated with severity in schizophrenia [15]. Fronto-striatal fMRI activity has been linked to cognition in healthy populations [16], patients with schizophrenia [17], and individuals with genetic high risk for schizophrenia [18]. In ventral striatum, nucleus accumbens (NAc) dysfunction has been heavily implicated in schizophrenia [19, 20]. Via the nucleus accumbens, the prefrontal cortex and hippocampal formation can influence striato-pallido-thalamo-cortical circuits involving the ventral pallidum, subthalamic nucleus, ventral tegmental area, substantia nigra and thalamic areas [21, 22].

Cognitive flexibility adapts goal-directed behaviour in the face of conflict or varying environmental demands and is a key component of the “executive control” processes that underpin everyday function and adaptive behaviour. Deficits in the ability to flexibly update behaviour are manifest in a wide range of neurological and psychiatric disorders, including autism, obsessive–compulsive disorder, major depressive disorder and schizophrenia [23–30] and are consistently linked to fronto-striatal dysfunction in patients [17, 28, 31–33]. Extensive research in rodents and non-human primates emphasises the major roles of fronto-striatal circuits in the ability to respond flexibly [34–42]. Conditional discrimination tasks have been suggested as a useful translational paradigm for assessing the cognitive inflexibility observed in neuropsychiatric disease [43]. In these tasks, animals are required to learn associations between arbitrary pairs of stimuli, or between arbitrary stimuli and responses. Crucially, successful performance requires that animals respond flexibly—making different responses in the presence of different cues. George and colleagues [44] have demonstrated direct evidence that conditional discrimination tasks engage frontal and meso-limbic dopaminergic systems, with dopamine increased in the prefrontal cortex and reduced in the nucleus accumbens region of the striatum in wildtype rats performing this task.

In the present work, we sought to quantify the neurophysiological and functional phenotypes associated with the structural abnormalities previously observed in *Cyfip1*^+/−^ rats. We used in vivo electrophysiological assays of distributed network coordination to examine whether these disrupted white matter tracks may lead to maladaptive fronto-striatal limbic coordination in the *Cyfip1*^+/−^ rats. We further hypothesised that the cognitive phenotype of *Cyfip1*^+/−^ rats would extend to a translational cognitive discrimination task known to recruit fronto-striatal circuitry.

## Methods

### Subjects

All procedures were conducted in accordance with the UK Animals (Scientific Procedures) Act, 1986 and with the approval of Cardiff University and University of Bristol ethics committees. This study used a total of 49 adult male, Long Evans rats (25 heterozygous knockouts [*Cyfip1*^+/−^], 24 wildtypes [WT]) from a novel line designed to recapitulate the low *CYFIP1* dosage caused by 15q11.2 CNV deletions in patients. The *Cyfip1* rat model was created by Cardiff University in collaboration with Horizon Discovery (St Louis, USA) using CRISPR–Cas9 targeting. CRISPR/Cas9 engineering of exon 7 implemented a 4 base-pair deletion in DNA of the *CYFIP1* gene on one of the chromosomes, causing a premature stop codon leading to reduced *CYFIP1* mRNA and *CYFIP1* protein in the brain, mimicking the human situation in the 15q11.2 CNV. Full information on the creation and validation of the rat model can be found in Silva, Haddon et al. [11].

Twenty-seven rats (14 WT, 13 *Cyfip1*^+/−^) were implanted with multisite Local Field Potential [LFP] recording electrodes; the remaining 22 rats (10 WT, 12 *Cyfip1*^+/−^) were trained on an instrumental conditional discrimination, see also Supplementary Table 1 for final n’s. These sample sizes were utilised on the basis of previous published research [11, 44, 45] and pilot studies. The animals were assigned to these groups in a non-blinded, random manner.

### Electrophysiology methods

#### Behaviour and surgery

Fourteen WT and 13 *Cyfip1*^+/−^ rats were implanted under isoflurane recovery anaesthesia with multisite LFP electrode arrays of 60 µm nichrome wire (A–M Systems, gold plated to impedance of 200–300 kkW at 1 kHz) targeting nucleus accumbens core [NAcC] (+1.6 mm anterior of bregma, +1.2 mm lateral of midline, −7.7 mm deep), nucleus accumbens shell [NAcS] (+1.6 mm, +1.2 mm, −6.6 mm), hippocampal dorsal CA1 [dCA1] (−3.2 mm, +2.2 mm, −2.2 mm), hippocampal ventral CA1 [vCA1] (−5.6 mm, +5.0 mm, −7.8 mm), prelimbic cortex [PRL] (+3.2 mm, +0.6 mm, −2.8 mm) and infralimbic cortex [IL] (+3.2 mm, +0.6 mm, −4.4 mm). Standard aseptic techniques and fluid therapy were used throughout surgery, with body temperature regulated using a heat-mat and rectal probe, while heart rate and blood oxygen concentration were monitored with a paw sensor (PhysioSuite, Kent Scientific). Eyes were shielded from surgery lights and kept hydrated with LacriLube (Allergan). Once the righting reflex had recovered, buprenorphine (0.025 mg/kg, Vetergesic) was administered subcutaneously and recovery diet gel and mashed rat chow were provided for three days, while water intake and weight gain were monitored. One animal was excluded from all analyses due to saturating noise during recording (WT = 14, *Cyfip1*^+/−^ = 12). A second animal was excluded due to a mis-targeted IL electrode. Thus, results for the analyses are shown for 14 WT and 11 *Cyfip1*^+/−^ rats.

#### Resting state LFP data

Starting 3 days after surgery, rats were habituated to regular handling and the recording room over 7 days prior to recordings. Resting state data were captured during a single 1 h recording session from each rat, during the light phase (local time 12:00–14:00). Electrophysiological data were acquired using Digital Lynx SX hardware (Neuralynx, MT), with a HS-36 headstage and ‘Litz’ tether. The tether was supported by a counterbalanced pulley system to enable free movement, with cameras monitoring behaviour at 720 × 576 pixel resolution, 30 frames per second. Ten minutes segments of resting-state (quiet wakefulness) data in the homecage were analysed, with sleep epochs excluded on the basis of cortical slow-wave and/or spindle activity and any high-amplitude movement artefacts removed. Local Field Potential data was recorded at 1017 Hz from frontal cortex (PRL/IL), ventral striatum (NAcC/NAcS), and hippocampus (dCA1 and vCA1).

#### Granger causality analysis

Granger causality analysis was performed with the multivariate Granger causality (MVGC) toolbox version 1.2 [46]. The MVGC approach estimates Granger causality from a vector autoregression (VAR) model of the neuronal time series data with Granger causality calculated using a state space-based approach [47]. LFP data were down-sampled to 200 Hz, notch filtered to remove 50 Hz line noise, de-meaned, and normalised across recording sites and animals. Data was divided into 120 epochs of 5 seconds for each animal, with epochs containing recording artefact or excess noise removed prior to analysis. The VAR model order (number of lags in the model) was determined using the Akaike information criterion (AIC). In these LFP data, the selected VAR model order ranged from 7 to 26. Each VAR model had an *R*^2^ > 30% and white residuals as assessed using the Durbin–Watson test (*p* < 0.05) with a mean model consistency of 84%. These values are consistent with the VAR models used to calculate Granger causality providing a robust description of the underlying neural data.

#### Post-mortem histology

Rats were terminally anaesthetised with sodium pentobarbital (60 mg/kg), and a positive 30 µA current was passed down each LFP wire for ~10 s to create a lesion at the tip. Rats were perfused transcardially with ~300 ml 0.9% saline, then with ~300 ml 4% paraformaldehyde in phosphate buffered saline. Coronal sections of 50 µm were cut with a freezing microtome and mounted on SuperFrost Plus slides (Thermo Scientific). Following drying, slides were Nissl stained with thionin, and lesions were identified using a digital camera linked to an optical microscope (DM100, Leica). Lesion sites were cross-checked with the Rat Brain Atlas [48], see Supplementary Fig. 1 for electrode placements.

### Behavioural methods

Animals were housed in groups of 2–4 males in cages of mixed genotypes. Age and weight ranges when the experiment began were 180–197 days (median = 200 days) and 565–819 g (median = 701 g). The room was temperature controlled (21 ± 2˚C) and on a 12-h light–dark schedule, with lights on from 07.00. Water was always available in home cages. Subjects’ weights were maintained at 85% of free-feeding weight to motivate participation in tasks for food rewards.

#### Apparatus

Training and testing took place in 8 operant chambers (30 cm wide × 24 cm deep × 21 cm high; Med Associates, St Albans, VT) with aluminium walls and a Perspex door, housed in sound-attenuating cabinets and arranged in a two-by-four array. Food pellets (45 mg; P. J. Noyes, Lancaster, NH) could be delivered into a recessed magazine located in the right wall of each chamber, with magazine access detected by infrared sensors. Two retractable levers could be inserted to the left and right of the magazine. Auditory stimuli consisted of a 2 kHz tone and a 10 Hz train of clicks delivered from speakers located in the ceiling

#### Procedure

*Pretraining*: All rats received sessions of magazine and lever press training, learning to retrieve pellets from the magazine, and to press both levers for reward. Animals that failed to collect all the rewards from the magazine or did not respond on both levers were removed from the experiment. Further details are provided in Supplementary Methods 1.

**Fig. 1:**
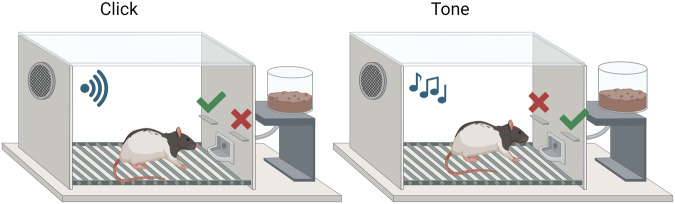
Graphical representation of the biconditional discrimination task. Rats were trained on two auditory stimuli (Tone and Clicker). For example, presses on the right lever during presentation of the Tone would lead to food, whereas left lever presses did not. In contrast, during presentations of the clicker, left lever presses resulted in reinforcement, whereas right lever presses led to nothing. Thus, as each auditory cue and lever is both associated with reward and no reward, (e.g. Tone → Right+, Tone → Left−, Click → Right−, Click → Left +) rats have to learn the specific cue-response combinations in order to maximise reward. Created with BioRender.com.

*Biconditional discrimination training*: Table 1 shows the experimental design for all animals in the behavioral cohort. Rats were trained for a total of 8 days on an auditory conditional instrumental discrimination, in which they received presentations of two auditory stimuli (Tone and Clicker), during which different lever-presses resulted in reinforcement (food pellets), counterbalanced across animals, a visual representation of the task is shown in Fig. 1. Animals were assigned to the different counterbalancing groups based on performance during lever press training. For example, presses on the right lever during presentation of the Tone would lead to food, whereas left lever presses did not. In contrast, during presentations of the clicker, left lever presses resulted in reinforcement, whereas right lever presses led to nothing. Thus, as each auditory cue and lever is both associated with reward and no reward, (e.g. Tone → Right+, Tone → Left−, Click → Right−, Click → Left+) rats have to learn the specific cue-response combinations in order to maximise reward. Learning about just one lever, or one cue, would result in a sub-optimal level of reward (i.e., they would only get rewarded during 50% of trials). Sessions consisted of 24 trials (12 of each trial type; A1/A2) with a variable ITI (range = 45–180 s, mean = 120 s). Stimulus presentations lasted 60 s, with reinforcement being unavailable during the first 10 s and available during the final 50 seconds on a random interval—15 s (RI15) schedule in which a reward becomes available on average every 15 s and the next lever press performed will result in delivery of the pellet. Both levers were presented during each trial and retracted during ITIs. Further details of the task can be found in George, Jenkins and Killcross and Haddon and Killcross [44, 49].

**Table 1:** Experimental Design.

Biconditional discrimination
Tone : Right lever press → +(Food), Left lever press → −(No food)
Click : Right lever press → −(No food), Left lever press → +(Food)

Auditory stimuli (Tone, Click) and levers (Left and right) were counterbalanced across animals.

## Results

### *Cyfip1* haploinsufficiency disrupts cortico-striatal network dynamics

To test the hypothesis that white matter changes observed previously in *Cyfip1*^+/−^ rats and 15q11.2del carriers are associated with disordered network activity, we examined resting-state functional connectivity between all recorded regions using unbiased Granger causality analyses.

**Fig. 2:**
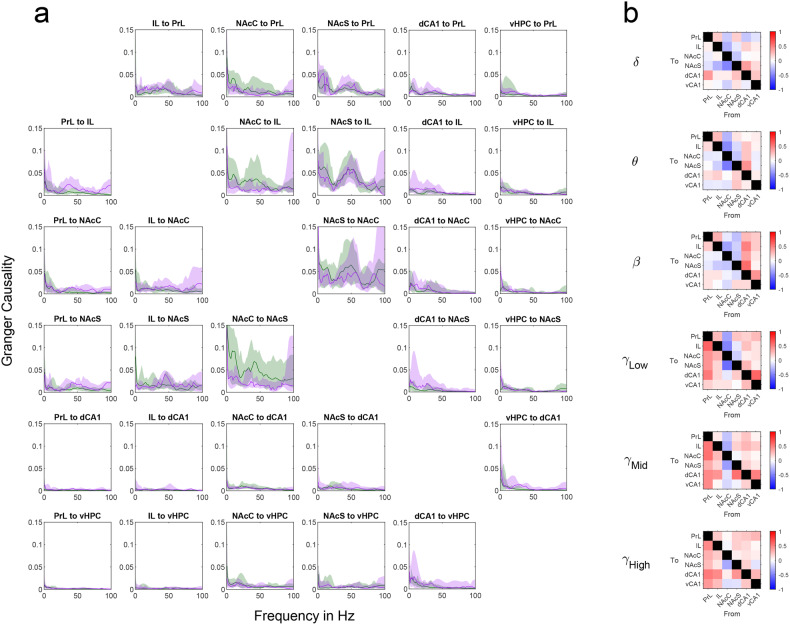
Cortico-striatal network dynamics. **a** Pairwise spectral Granger causality for WT and *Cyfip1*^*+/−*^ *animals*. Median pairwise spectral Granger causality is shown in in green for WT animals (*n* = 14) and purple for *Cyfip1*^+/−^ animals (*n* = 11). The shaded area range surrounding each line represents the interquartile range. For each plot y axis shows Granger causality, while the *x*-axis shows frequency in Hz. For each region pair the Granger causality in the opposing direction is in the corresponding position relative to the diagonal mirror line. **b** Comparison of band-limited pairwise Granger causality between WT and *Cyfip1*^+/−^ animals. The difference between Granger causality is shown between WT and *Cyfip1*^+/−^ animals for each combination of frequency band and directional connection. The scores on the colourmap represented W statistic from the Wilcoxon rank sum test scaled to the range [−1,1]. Blue squares represent comparisons where there was lower Granger causality in the HET group relative to WT; red squares represent higher values of Granger causality in the HET group relative to WT; and white indicates no difference. None of these findings remained significant following FDR correction.

Wideband spectral pairwise conditional Granger causality was calculated over 0–100 Hz frequency range, see Fig. 2. Band-limited pairwise conditional Granger causality was then quantified as the integral of the full band Granger causality over frequency bands thought to reflect limbic-cortical and striatal-cortical interactions: delta (δ, 2–4 Hz), theta (θ, 6–10 Hz), beta (β, 15–25 Hz), low gamma (γlow, 35–45 Hz), mid gamma (γmid, 55–75 Hz), and high gamma (γhigh, 70–90 Hz) [46].

As Granger causality follows a non-parametric distribution, we used the two-sided Wilcoxon rank sum test for independent samples to test for statistical differences in the band limited Granger causality between WT (*n* = 14) and *Cyfip1*^+/−^ (*n* = 11) animals, Fig. 2b. To visualise the differences in Granger causality between WT and *Cyfip1*^+/−^ animals, the W statistic of the Wilcoxon rank sum test was scaled to the range [−1,1] with negative numbers representing lower values of Granger causality in the *Cyfip1*^+/−^ group relative to WT, and positive numbers representing higher values of Granger causality in the *Cyfip1*+*/−* group relative to WT. This approach to visualisation was adapted from that of Barnett et al. [50]. Using this approach there appears to be a strong trend for higher Granger causality from the nucleus accumbens Core (NAcC) to all regions in WT rats, that was evident across all frequency bands. In contrast, *Cyfip1*^+/−^ rats have higher Granger causality originating from the Prelimbic cortex and hippocampus. Despite these trends, no significant differences were observed between WT and *Cyfip1*^+/−^- animals once corrected for false discovery rate [51, 52].

**Fig. 3:**
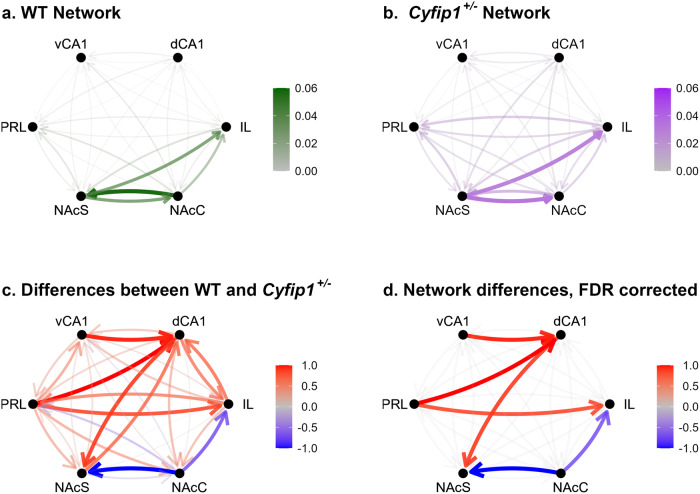
Comparison of Granger causality between WT and *Cyfip1*^+/−^ animals across all frequencies. The median Granger causality scores for **a** WT and **b** *Cyfip1*^+/−^ animals. WT values are shown in green and *Cyfip1*^+/−^ values are shown in purple. Thicker lines represent higher Granger scores. **c** The difference between Granger causality is shown between WT and *Cyfip1*^+/−^ animals. The scores on the network plot represent the W statistic from the Wilcoxon rank sum tests scaled to the range [−1,1]. Blue lines represent comparisons where there was lower Granger causality in the HET group relative to WT; red lines represent higher values of Granger causality in the HET group relative to WT; and grey indicates no difference. **d** Comparisons that remained significant following FDR correction.

Structural, white-matter abnormalities *Cyfip1*^+/−^ rats and 15q11.2del carriers could, in principle, impact inter-regional communication across a range of carrier frequencies. To capture net differences in functional connectivity across the network, we therefore quantified the differences between WT and *Cyfip1*^+/−^ Granger causality across all the frequency bands between 0–100 Hz [final *n* = rat*frequency band: WT *n* = 84, *Cyfip1*^+/−^ *n* = 66]. The median Granger scores for each pairing in WTs and *Cyfip1*^+/−^ animals are shown in network connectivity plots (Fig. 3a, b respectively). WTs had stronger Granger causality emanating from a circuit involving the nucleus accumbens (core and shell), and the infralimbic cortex. In contrast, *Cyfip1*^+/−^ animals had weaker Granger causality overall, plus connectivity was more diffuse than in WTs. These broadband Granger causality values were compared using Wilcoxon rank sum and the subsequent W values are shown in Fig. 3c. Six of these pairings survived correction for false discovery rate (Fig. 3d). Stronger Granger causality was observed from the nucleus accumbens core to both the NAc shell (*W* = 1636, *p* < 0.001) and infralimbic cortex (*W* = 1882, *p* < 0.01) in WTs compared to *Cyfip1*^+/−^ animals. In contrast, stronger Granger causality was observed in pairings emanating from the prelimbic cortex to the dCA1 (*W* = 3717, *p* < 0.01) and IL (*W* = 3570, *p* < 0.05) in *Cyfip1*^+/−^ animals. *Cyfip1*^+/−^ animals also showed stronger hippocampal drive between the dCA1 to the NAcS (*W* = 3605, *p* < 0.05), and from the vCA1 and dCA1 (*W* = 3678, *p* < 0.01) compared to WT animals, suggesting more disordered fronto-striatal-limbic network connectivity in the *Cyfip1*^+/−^ animals. Full statistics can be found in Supplementary Table 2. Next, we sought to examine whether this disrupted resting-state network activity translated into impaired performance on a behavioural task known to rely on cortico-striatal circuitry.

### Low dosage of *Cyfip1* impairs cognitive flexibility on a conditional discrimination task

All subjects progressed from magazine training, with 20 (9 WT, 11 *Cyfip1*^+/−^) progressing to RI15 lever-press training (1 *Cyfip1*^+/−^ and 1 WT did not reach criterion). These final n are in keeping with previous studies using conditional discrimination tasks [49]. There were no genotype-dependent differences in performance during any pre-training phases (Supplementary Table 3**)**. Similarly, no significant differences between genotype on magazine entry behaviour or rewards earned were observed during conditional discrimination training (Supplementary Figs. 2 and 3 respectively).

**Fig. 4:**
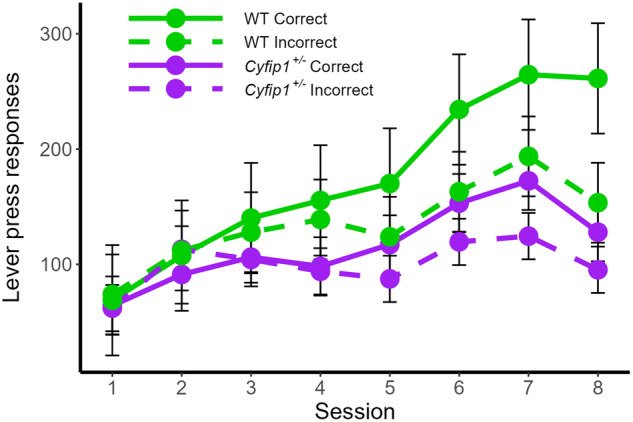
Conditional discrimination performance. Acquisition of the auditory discrimination is impaired in *Cyfip1*^+/−^ rats: producing fewer correct responses compared to WT rats. Mean ± SEM, WT *n* = 9, *Cyfip1*^+/−^ *n* = 11.

Preliminary analysis of instrumental discrimination performance during the two auditory conditional cues (Supplementary Fig. 4**)** revealed a significant effect of TRIAL type (Tone and Click) on instrumental performance: such that both correct and incorrect lever press responding was elevated during presentations of the Clicker compared to the Tone trials. This difference was most pronounced from session 5 onwards. No effects or interactions with genotype were observed, hence for clarity, responses to the two stimuli were totalled to produce an overall measure of biconditional discrimination task performance in the two groups as shown in Fig. 4. WT rats successfully acquired the biconditional discrimination, producing more correct than incorrect responses by the end of training. In contrast, the performance of *Cyfip1*^+/−^ rats was disrupted: although they produced more correct than incorrect responses to the biconditional stimuli, they were impaired compared to WT rats and produced fewer instrumental responses overall.

A mixed ANOVA with a between-subjects factor of GENOTYPE (WT, *Cyfip1*^+/−^) and a within-subjects factor of SESSION (1–8) and LEVER (correct, incorrect) revealed a significant GENOTYPE × SESSION × LEVER interaction *F*(7, 126) = 2.560 *p* = 0.017. Simple effects analyses revealed a GENOTYPE × LEVER interaction during the final session of training (session 8) [F(1, 144) = 16.637, *p* < 0.001]. An effect of LEVER was observed in both WT [*F*(1, 144) = 68.393, *p* < 0.001] and *Cyfip1*^+/−^ rats [*F*(1, 144) = 6.258, *p* = 0.014] such that all animals produced more correct than incorrect responses during the last session of training. *Cyfip1*^+/−^ rats, however, produced significantly fewer responses on the correct lever than WT rats [*F*(1, 288) = 9.246, *p* = 0.003].

### *Cyfip1*^+/−^ rats demonstrate inflexible responding

**Fig. 5:**
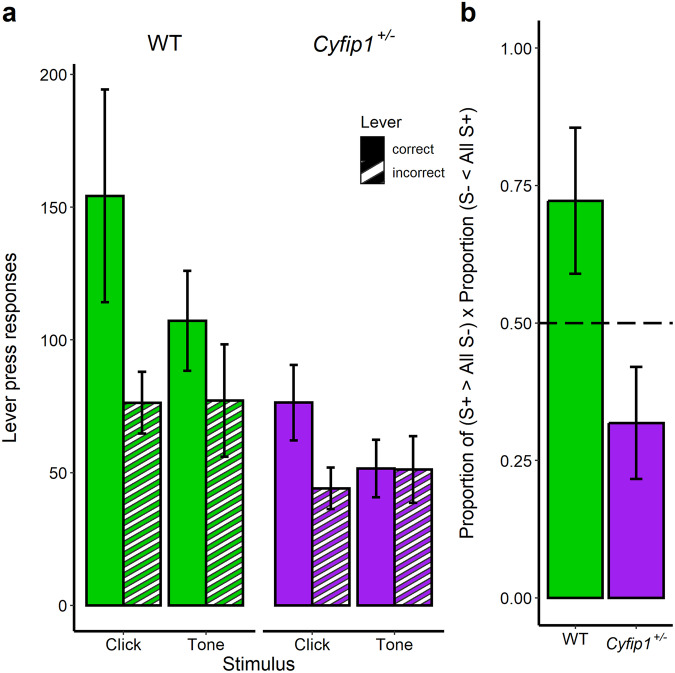
Behavioural flexibility. **a** WT rats (green bars) show correct responding to both elements (cues) of the biconditional discrimination during the final session of biconditional training, whereas *Cyfip1*^+/−^ rats (purple bars) show correct responding to only one cue (the clicker). Mean ± SEM, WT *n* = 9, Cyfip1+/− *n* = 11. **b** WT rats show successful learning about the complete biconditional task whereas *Cyfip1*^+/−^ rats do not. Discrimination ratio reflects the Proportion of (S+ > All S−) × Proportion (S− < All S+) and provides a score between 0 and 1, where 0 is No discrimination and 1 reflects perfect correct responding to both cues. The dotted line represents the value that might be expected if animals were responding according to chance, or to one of the stimuli alone. Mean ± SEM, WT *n* = 9, *Cyfip1*^+/−^ *n* = 11.

Biconditional performance was further analysed by investigating responses to the different auditory cues (Tone and Click) during the final session of training (session 8, see Fig. 5a). While WT rats showed correct responding to both the Clicker and the Tone, *Cyfip1*^+/−^ rats only appeared to respond correctly to the Clicker, responding equally on the correct and incorrect levers to the Tone. A mixed ANOVA with factors of GENOTYPE, TRIAL TYPE and LEVER revealed a significant GENOTYPE × LEVER interaction [*F*(1,18) = 7.266, *p* = 0.015]. Simple effects analysis revealed an effect of LEVER for WT [*F*(1,18) = 29.870, *p* < 0.001] but not *Cyfip1*^+/−^ rats [*F*(1,18) = 2.733, *p* = 0.116, n.s.], such that WT rats responded more to the correct lever than the incorrect lever. A significant effect of GENOTYPE was observed on correct responses [*F*(1,36) = 7.528, *p* = 0.009] but not responses on the incorrect lever [*F*(1,36) = 1.431, *p* = 0.240, n.s.].

As the ANOVA analysis was based on group means it is theoretically possible that some WT animals, like the *Cyfip1*^+/−^ rats, did not truly learn the biconditional discrimination, instead learning only one component of the discrimination. If the cue-response associations learned by different individuals were equally distributed across the two cues, group averaging could obfuscate this result. We therefore adopted a stricter measure based on a discrimination ratio score, which takes into account responding to both cues. This measure was adapted from similar approaches reported previously [53, 54] and involves calculating Proportion of (S+ > All S−) × Proportion (S− < All S+) and provides a score between 0 and 1, where 0 is no discrimination and 1 reflects perfect correct responding to both cues. Figure 5b shows these scores for both WT and *Cyfip1*^+/−^ rats during the final session of training, again demonstrating that WT rats performed significantly more accurately than *Cyfip1*^+/−^ rats [One way ANOVA revealed an effect of GENOTYPE *F*(1, 18) = 5.403, *p* = 0.032] with WT rats showing a score that was significantly above chance responding and *Cyfip1*^+/−^ rats responding as if they have only learned about one cue of the biconditional discrimination task. That is, *Cyfip1*^+/−^ animals were unable to respond flexibly to the different conditional cues. In contrast, WT rats responded in a cue-directed manner, responding correctly to both conditional auditory stimuli.

## Discussion

Previous research has reported changes to white matter in the low dosage *Cyfip1* rat model, most prominently in corpus callosum [11], a finding consistent with reports in mouse models [10] and 15q11.2(BP1–2)del copy number variation (CNV) carriers [12]. In the rat model, these changes in white matter have been linked to thinning of the myelin sheath on axons, independent of changes in the number or diameter of axons [11]. Axon-myelin perturbations can have marked effects on brain network activity as a consequence of changes to the temporal coherence of action potential integration across different brain regions required for adaptive brain functioning [55, 56]. Using multi-site LFP recording, we have shown that *Cyfip1* haploinsufficiency in the rat results in abnormal functional connectivity between key fronto-striatal regions that are necessary for behavioural flexibility and cognitive control. *Cyfip1*^+/−^ rats were also unable to successfully learn a conditional discrimination task known to recruit the same circuitry.

Using resting-state LFP recordings, we show that *Cyfip1*^+/−^ haploinsufficiency was associated with a functional network in which the influence of the nucleus accumbens was diminished, and connectivity involving frontal cortex and hippocampus was weaker and more widespread. Broadly, these findings are consistent with reports in neuropsychiatric disorders of brain network dysconnectivity and failures of neural integration [57–59] insofar as many studies have indicated cortico-striatal network dysregulation is linked to psychotic symptoms [60], symptom severity [61], and response to antipsychotic treatment [62]. Moreover, the striatum has been highlighted as playing an important role in the pathogenesis of the cognitive symptoms of schizophrenia [63].

Alongside the disordered network dynamics centred on the nucleus accumbens and its connections, *Cyfip1*^+/−^ animals proved prone to behavioural inflexibility in a conditional discrimination paradigm, known to engage fronto-striatal circuitry [44]. *Cyfip1* heterozygous rats showed a selective deficit in responding flexibly during the conditional discrimination task - being able to respond correctly during only one of the cue types, responding incorrectly when the other cue was presented. In contrast, WT rats responded correctly to both conditional cues. *Cyfip1*^+/−^ rats were, however, unimpaired on the more straightforward elements of the task. They were able to learn to approach the magazine, and press levers for reward during pretraining to a similar degree to WT rats and could learn a single stimulus-response association. Previous animal studies have revealed that performance on conditional discrimination tasks is dependent upon the intact functioning of the nucleus accumbens [64, 65]. The nucleus accumbens is strongly linked to both behavioural flexibility and inhibitory control, including goal-directed and habitual behaviour [66–68]. Lesions to the nucleus accumbens can produce perseverative responding, impair reversal learning, and reduce behavioural switching [69–71].

Dysfunctional connectivity in fronto-striatal circuitry is supported by evidence in 15q11.2 CNV deletion carriers with widespread differences in brain morphology seen throughout the cortex, including the frontal and cingulate cortices. In contrast, subcortical differences appear restricted to decreased volume in the accumbens. These differences in frontal and accumbens morphology were associated with reduced cognitive flexibility on the Trial Making Task (B) [72, 73]. Aberrant brain connectivity has been observed using resting-state MEG in adults with a wide range of neurodevelopmental CNVs including 15q11.2 [74], although primary abnormalities involved decreased connectivity between occipital, temporal, and parietal areas and were only measured in 7 15q11.2 deletion carriers. In animals, Babbs et al. [75] found that maternally inherited *Cyfip1* haploinsufficient mice have reduced *Cyfip1* gene expression in the nucleus accumbens and an increase in compulsive-like behaviours. Moreover, our results are in keeping with deficits in the ability to flexibly update behaviour observed in neurological and psychiatric disorders such as Parkinson’s Disease, schizophrenia and autism [23–31].

In addition to the reduced accumbens connectivity seen in *Cyfip1*^+/−^ rats, increased input from the prefrontal cortex and hippocampus—both regions that are linked with cognitive flexibility [37, 76] and neurodevelopmental disease [77, 78]—was also observed. The prefrontal cortex is extensively linked to flexible behaviour and executive function. For example, Piao et al. [79] found that abnormal hyperactivity in the medial PFC combined with dysfunction in the nucleus accumbens core associated with long term deficits in executive function in rats exposed to prolonged stress. Mice with Cyfip1 haploinsufficiency have been found to have a reduced number of synapses in the hippocampus [2], and have abnormal postnatal hippocampal neurogenesis [80]. Hippocampal neurogenesis-mediated inhibition has been proposed as a mechanism to reduce memory interference—enabling cognitive flexibility in tasks such as reversal learning [76], which we have previously shown is sensitive to reductions of *Cyfip1* in the rat [11]. The ability of the hippocampus to promote response strategies is proposed to be partly supported via projections from the hippocampus to the NAc shell [81]. Hippocampal projections to the NAc have also been hypothesised to play a role in behavioural flexibility during latent inhibition [82, 83]. Hence, changes in hippocampal structure and connectivity could also contribute to the failure in conditional discrimination performance seen in *Cyfip1*^+/−^ rats.

Previous studies, in *Cyfip1* haploinsufficient animal models as well as 15q11.2 deletion CNV carriers have all reported marked changes to white matter in corpus callosum microstructure [11–13]. Increased integrity of white matter in the corpus callosum and internal capsule has been found to be positively associated with activation in the nucleus accumbens [84]. It is well documented that the nucleus accumbens receives innervation from multiple brain regions including the prefrontal cortex and hippocampus [85, 86]. Accumbens neurons require activation from more than one source to reach threshold [87, 88] - integrating limbic and cortical innervations depending on the intensity and timing of inputs [89]. The corpus callosum carries white matter bundles containing axons from prefrontal cortex and striatal regions [90]. Hence the changes in callosal white matter microstructure showing reduced myelination of nerve fibres previously reported in *Cyfip1*^+/−^ rats [11] may differentially constrain information transfer to and from the accumbens, resulting in the dysfunctional connectivity observed in this current study. However, the stability of fronto-striatal integrity was not quantified in our experiments; longitudinal studies quantifying structural and/or functional connectivity across days and different neurodevelopmental timepoints would be one useful next step.

Given the widespread changes in functional connectivity between brain regions, it is possible that other functional impairments – for example in auditory perception and/or motor responding—may contribute to behavioral phenotypes in *Cyfip1*^+/−^ rats. No differences, however, were observed in motor responding (magazine entries or lever press responses) during pretraining, or magazine entries during training on the conditional task. Moreover, no differences were observed in magazine entry responses to the different auditory cues during conditional discrimination training (Supplementary Table 4), suggesting both cues were equally able to elicit a Pavlovian approach response. Hence, any contribution of these factors to conditional performance is likely to involve a more complex situation in which instrumental behaviour is modulated by auditory perception such as recently reported in mice following manipulation of nigrostriatal dopamine pathways [91]. It is also worth noting that there are no consistent reports of deficits in either hearing or perception in individuals with 15q11.2del.

The animals in the current study were trained blind to genotype for eight days on the discrimination task—which we have previously shown to be long enough for WT rats to successfully acquire the discrimination [44, 49]. It is possible that with extensive training and/or more salient auditory cues some of the *Cyfip1*^+/−^ rats may be able to successfully learn the full conditional discrimination. For example, some *Cyfip1*^+/−^ were able to complete a reversal learning task in which performance to criteria rather than number of sessions of training dictated how long animals were trained for [11]. Nevertheless, this does and would not negate from the finding that *Cyfip1*^+/−^ rats are impaired on the conditional discrimination task in the current study.

Converging evidence suggests a crucial role for dopamine in the aetiology of neurodevelopmental disorders such as schizophrenia [92, 93]. The conditional discrimination task used here has been shown to recruit meso-limbic dopaminergic systems in the frontal cortex and nucleus accumbens [44], and is highly sensitive to dopamine dysfunction [94, 95]. Moreover, cortico-striatal synchrony is disrupted by treatment with phencyclidine [96], and anti-psychotic drugs that act on dopamine also alter the functional connectivity between these regions and the hippocampus [97]. Recent work comparing a *Cyfip1* knockout and overexpressing mouse models by Kim and colleagues [98] has shown a key role for *Cyfip1* in synaptic function: translation of mRNAs encoding the NMDA receptor complex (GRIN2A and GRIN2B) and associated scaffold proteins (HOMER1, SHANK2, PSD95) were significantly increased in the hippocampus of knockout mice, but were reduced in the *Cyfip1* overexpression model. Moreover, this loss of *Cyfip1* may drive overactive NMDAR signalling, hippocampal hyperactivity and aberrant drive of dopamine release; behavioural deficits in the *Cyfip1* knockout and overexpressing lines could be ameliorated by pharmacological treatment with NMDAR antagonists and agonists, respectively. Our results demonstrate that the impacts of reduced *Cyfip1* dosage extend beyond the hippocampus, though age- and region-restricted knockdown may help to disentangle causal circuit mechanisms. Nevertheless, taken together, these observations suggest that deficits in cortico-limbic network activity seen in *Cyfip1*^+/−^ rats may be linked to dopamine dysfunction.

In conclusion, we have employed a novel rat model of *Cyfip1* haploinsufficiency to probe the electrophysiological and behavioural fronto-striatal mechanisms underlying the enhanced risk for neuropsychiatric disease linked to the 15q11.2 BP1-BP2 deletion. Taking advantage of complex cognitive testing and network recordings from multiple brain regions feasible in rats, we demonstrate the first evidence for widespread functional disturbances to fronto-striatal functional network connectivity in an animal model, together with a highly specific inability to learn a cognitive discrimination task known to recruit the same regions. This extends the idea that reduced myelination in *Cyfip1*+*/−* rats results in impaired brain network function, which in turn leads to cognitive impairment consistent with behavioural deficits seen in neurodevelopmental disorders such as autism and schizophrenia. This study establishes a useful framework that encompasses the widespread cognitive and brain network dysfunction seen in neuropsychiatric diseases, combined with a novel animal model which can thus be used to design and test novel interventions for treatment in CNV carriers, and other neuropsychiatric patients.

### Supplementary information

Supplemental Material

## Data Availability

The source data underlying Figs. 2–5 and Supplementary Figs. 1–4 will be made available at https://osf.io/uhv5z/.
