# Supplementary material for: Linking haploinsufficiency of the autism- and schizophrenia-associated gene Cyfip1 with striatal-limbic-cortical network dysfunction and cognitive inflexibility
Source: Transl Psychiatry. 2024 Jun 14;14:256. doi: 10.1038/s41398-024-02969-x (PMC11178837; doi:10.1038/s41398-024-02969-x)
Supplement: Supplementary file 1 — Supplemental Material [file 41398_2024_2969_MOESM1_ESM.docx]

Supplementary Material

|  | WT final n *[Excluded]* | *Cyfip1*^+/-^ final n *[Excluded]* |
| --- | --- | --- |
| Behavioural experiment | 10 *[1]*^a^ | 12*[1]* ^a^ |
| Electrophysiological recordings | 14*[0]* | 11*[2]* ^b^ |

**Supplementary Table 1. Final n for behavioural and electrophysiological experiments.**

^a^ Animals excluded as failed to reach criterion during lever press training. ^b^ One animal was excluded from electrophysiological recording data analysis due to a mistargeted recording electrode, the other due to noise contaminating the local field potential signal.


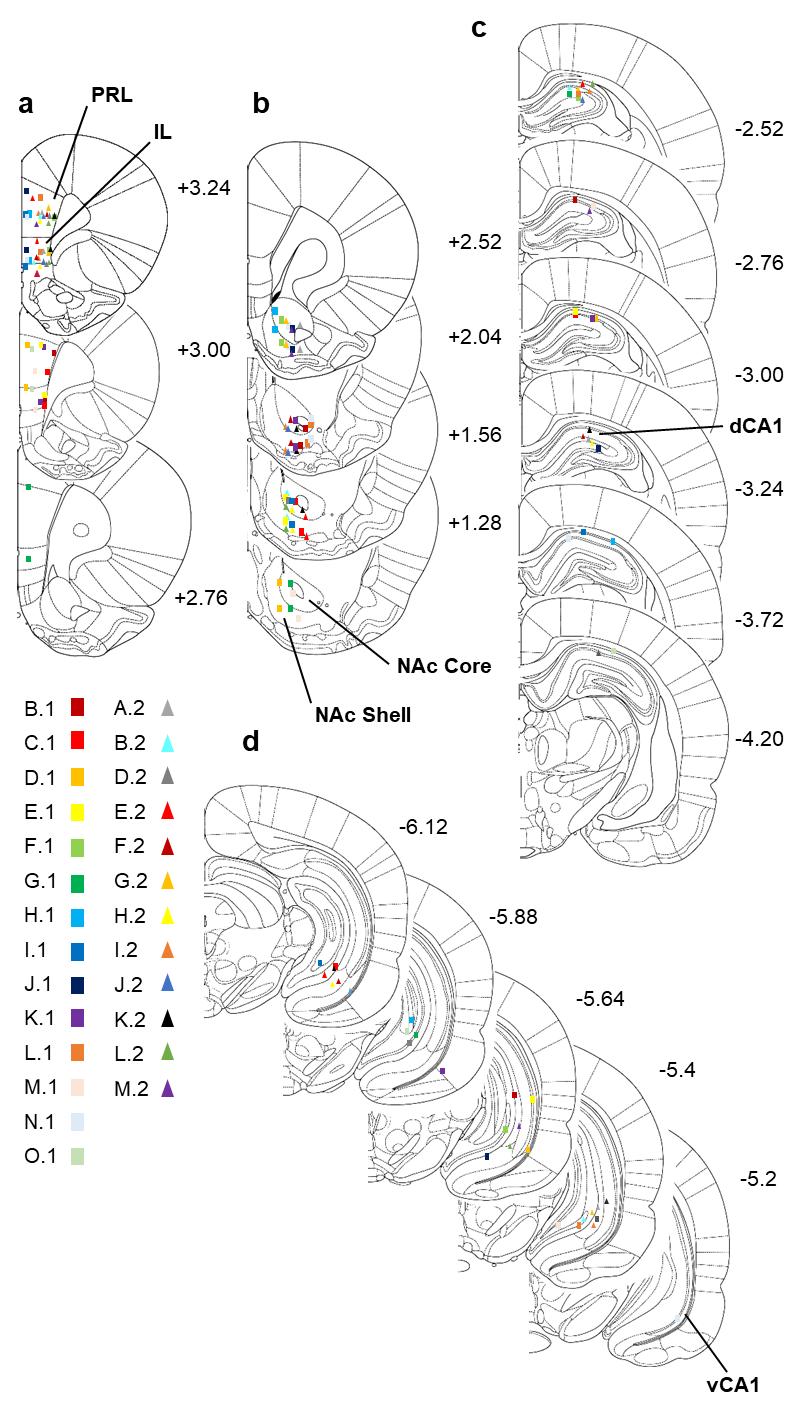


**Supplementary Figure 1. Histological verification of electrode placement. Serial section diagrams of structures with the position of electrodes from each rat indicated with coloured squares or triangles. Distances from bregma in mm shown on right. Figures taken from the Rat Atlas (Paxinos & Watson, 2007).**

Supplementary Methods 1. PRETRAINING : All rats received sessions of magazine training, learning to retrieve both pellets from the magazine. House lights were illuminated for the duration of each session and throughout all subsequent experimental sessions. Magazine training sessions lasted for 24 min, and rats received reward approximately every 120 s. Animals progressed to Lever Press Training when no pellets were left in the magazine when the session finished. During each of these sessions, the rats received 12 lever presentations (6 each of the right and the left lever, in random sequence), each lasting for 60 s and with an inter-lever interval of 60 s. On the first day of lever press training, rats were rewarded on a continuous reinforcement schedule in which every lever press resulted in reward. This was altered on subsequent sessions to a random interval 15 (RI15) schedule, such that reward becomes available on average once in a 15 s period and the next lever press response will lead to delivery of a reward. This RI15 schedule remained in place for the remainder of the experiment. Animals proceeded to biconditional discrimination training if they were responding similarly on both levers at the end of RI15 training. All subjects progressed from magazine training, with 20 (9 WT, 11 *Cyfip1*^+/-^) progressing to RI15 lever-press training (1 *Cyfip1*^+/-^ and 1 WT did not reach criterion). These final n are in keeping with previous studies using conditional discrimination tasks^49^.

| **Source** | **Target** | **WT median**  **(Inter-quartile range)** | ***Cyfip1^+/-^* median (Inter-quartile range)** | **W** | **p-value** | **FDR** |
| --- | --- | --- | --- | --- | --- | --- |
| PRL | IL | 0.0057 (0.003 - 0.0161) | 0.0125 (0.0063 - 0.0311) | 3570 | 0.00253 ** | 0.0152 * |
| PRL | NAcC | 0.0041 (0.0018 - 0.0159) | 0.0105 (0.0027 - 0.0198) | 3203 | 0.103 | 0.2852 |
| PRL | NAcS | 0.0074 (0.0031 - 0.0183) | 0.0096 (0.0049 - 0.0259) | 3169 | 0.133 | 0.342 |
| PRL | dCA1 | 0.0013 (6e-04 - 0.0031) | 0.0033 (0.0014 - 0.0073) | 3717 | 0.000349 *** | 0.0063 ** |
| PRL | vCA1 | 0.0014 (5e-04 - 0.004) | 0.002 (0.001 - 0.0047) | 3226 | 0.086 | 0.258 |
| IL | PRL | 0.0079 (0.0035 - 0.0225) | 0.0158 (0.007 - 0.0277) | 3372 | 0.0232 * | 0.0835 |
| IL | NAcC | 0.008 (0.0035 - 0.0176) | 0.0111 (0.0044 - 0.026) | 3129 | 0.177 | 0.396 |
| IL | NAcS | 0.0154 (0.0055 - 0.0259) | 0.014 (0.0065 - 0.0263) | 2682 | 0.735 | 0.945 |
| IL | dCA1 | 0.0015 (7e-04 - 0.0034) | 0.0025 (0.0012 - 0.0053) | 3434 | 0.0123 * | 0.0554 |
| IL | vCA1 | 0.002 (8e-04 - 0.0051) | 0.0024 (0.0011 - 0.0052) | 2906 | 0.613 | 0.8488 |
| NAcC | PRL | 0.0135 (0.0053 - 0.0446) | 0.01 (0.0039 - 0.0214) | 2279 | 0.0622 | 0.2036 |
| NAcC | IL | 0.0265 (0.0121 - 0.0583) | 0.0134 (0.0065 - 0.0304) | 1882 | 0.000758 *** | 0.0068 ** |
| NAcC | NAcS | 0.0567 (0.0189 - 0.1065) | 0.0198 (0.0092 - 0.041) | 1636 | 1.71e-05 **** | 6e-04 *** |
| NAcC | dCA1 | 0.0051 (0.002 - 0.0121) | 0.0053 (0.003 - 0.008) | 2789 | 0.95 | 1 |
| NAcC | vCA1 | 0.0096 (0.0035 - 0.018) | 0.0064 (0.0037 - 0.0123) | 2489 | 0.285 | 0.4937 |
| NAcS | PRL | 0.0126 (0.006 - 0.0308) | 0.0144 (0.006 - 0.033) | 2933 | 0.543 | 0.7819 |
| NAcS | IL | 0.0391 (0.0142 - 0.0678) | 0.0301 (0.0174 - 0.0494) | 2609 | 0.538 | 0.7819 |
| NAcS | NAcC | 0.0397 (0.019 - 0.0734) | 0.0317 (0.0155 - 0.0593) | 2423 | 0.187 | 0.396 |
| NAcS | dCA1 | 0.0033 (0.0013 - 0.0085) | 0.0059 (0.0026 - 0.0125) | 3448 | 0.0105 * | 0.054 |
| NAcS | vCA1 | 0.004 (0.0016 - 0.0182) | 0.0063 (0.0021 - 0.0133) | 2940 | 0.526 | 0.7819 |
| dCA1 | PRL | 0.0068 (0.0026 - 0.0135) | 0.0059 (0.0037 - 0.0177) | 3005 | 0.379 | 0.6202 |
| dCA1 | IL | 0.0048 (0.0016 - 0.0114) | 0.0072 (0.0023 - 0.0267) | 3385 | 0.0204 * | 0.0816 |
| dCA1 | NAcC | 0.0059 (9e-04 - 0.0169) | 0.0092 (0.0015 - 0.027) | 3141 | 0.163 | 0.3912 |
| dCA1 | NAcS | 0.0028 (6e-04 - 0.0112) | 0.0108 (0.0021 - 0.027) | 3605 | 0.00162 ** | 0.0117 * |
| dCA1 | vCA1 | 0.0067 (0.0019 - 0.0147) | 0.0069 (0.0028 - 0.027) | 3078 | 0.247 | 0.468 |
| vCA1 | PRL | 0.0027 (0.0011 - 0.0087) | 0.0043 (0.0017 - 0.0102) | 3053 | 0.288 | 0.4937 |
| vCA1 | IL | 0.0037 (0.0014 - 0.0128) | 0.0054 (0.0022 - 0.0114) | 3092 | 0.226 | 0.452 |
| vCA1 | NAcC | 0.0036 (0.0013 - 0.0156) | 0.0037 (0.0016 - 0.011) | 2849 | 0.772 | 0.9583 |
| vCA1 | NAcS | 0.0054 (0.0011 - 0.0123) | 0.0039 (0.0021 - 0.0092) | 2870 | 0.712 | 0.945 |
| vCA1 | dCA1 | 0.0024 (9e-04 - 0.0083) | 0.0074 (0.0024 - 0.0221) | 3678 | 0.000607 *** | 0.0068 ** |

**Supplementary Table 2. Comparison of Granger Scores between WT and Cyfip1^+/-^ animals across all frequency bands. * p<0.05, ** p<0.01, *** p<0.001.**

| Measure | WT Mean (SEM) | *Cyfip1^+/-^* Mean (SEM) |  | F value (d.f.) | p-value |
| --- | --- | --- | --- | --- | --- |
| Magazine Entries | 275.2 (+/- 33.77) | 246.99 (+/- 39.6) |  | 0.251 (1,18) | 0.621 |
| Lever presses | 170.74 (+/- 17.51) | 167.18 (+/- 23.21) |  | 1.043 (1,18) | 0.322 |
| Rewards | 21.71 (+/- 4.33) | 16.98 (+/- 3.41) |  | 0.680 (1,18) | 0.418 |

**Supplementary Table 3. Comparison of behavioural measures during pretraining of lever press responses. No effects of genotype were observed on any of the behavioural measures. Final n: WT = 9, Cyfip1+/- = 11.**

**Supplementary Figure 2. Magazine entries during biconditional discrimination training in WT and Cyfip1^+/-^. Data represent Mean ± SEM, Final n: WT = 9, Cyfip1+/- = 11.** A mixed ANOVA with a between-subjects factor of GENOTYPE (WT, *Cyfip1*^+/-^) and a within-subjects factor of SESSION (1-8) revealed a significant effect of SESSION F(7, 126) = 6.452, p < 0.001, but no main effect of GENOTYPE or interaction.

**Supplementary Figure 3. Rewards earned during biconditional discrimination training in WT and Cyfip1^+/-^. Data represent Mean ± SEM, Final n: WT = 9, Cyfip1+/- = 11.** A mixed ANOVA with a between-subjects factor of GENOTYPE (WT, *Cyfip1*^+/-^) and a within-subjects factor of SESSION (1-8) revealed a significant effect of SESSION F(7, 126) = 25.378 p < 0.001, but no main effect of GENOTYPE or interaction.

**Supplementary Figure 4. Lever press responding during a. Tone and b. Click trials in WT and Cyfip1+/- rats. Data represent Mean ± SEM, Final n’s: WT = 9, Cyfip1+/- = 11**

Both WT and *Cyfip1*^+/-^ rats responded more (both correct and incorrect response) on Click trials compared to Tone trials. Correct responding to Click trials was observed from session 5 onwards in WT rats, but only emerged in session 8 in *Cyfip1*^+/-^ rats. Initial responding to the Tone was incorrect in WTs (a natural consequence of the Click – lever association being learned first). Correct responding during Tone trials was seen from session 6 in WTs, but no difference between levers was observed in *Cyfip1*^+/-^ rats.

A mixed ANOVA with a between-subjects factor of GENOTYPE (WT, *Cyfip1*^+/-^) and a within-subjects factor of TRIAL (Tone, Click), SESSION (1-8) and LEVER (correct, incorrect) revealed a significant SESSION x TRIAL (F(7, 126) = 2.560 p = 0.017) interaction such that lever press responding (both correct and incorrect) was greater during Click compared to Tone trials from session 5 onwards (Min F ( 1, 144)= 6.600 p=0.011) . A GENOTYPE x SESSION x LEVER (F(7, 126) = 2.916 p = 0.007) interaction was also observed, but no other significant effects or interactions with TRIAL type were seen [GENOTYPE X TRIAL X SESSION approached significance F(1, 18) =3.4 p = 0.082].

| Magazine entries | WT Mean (SEM) | *Cyfip1^+/-^* Mean (SEM) |  | F value (d.f.) | p-value |
| --- | --- | --- | --- | --- | --- |
| Tone trials | 135.71 ((+/- 20.33) | 129.93 (+/- 24.44) |  | 0.031 (1,18) | 0.861 |
| Click trials | 171.54 (+/- 23.86) | 137.53 (+/- 22.89) |  | 1.045 (1,18) | 0.321 |

**Supplementary Table 4. Comparison of magazine entry responses to the auditory cues during biconditional discrimination training. No effects of genotype were observed. Final n: WT = 9, Cyfip1+/- = 11.**
